# Synthesis and Anticancer Evaluation of New Indole-Based Tyrphostin Derivatives and Their (*p*-Cymene)dichloridoruthenium(II) Complexes

**DOI:** 10.3390/ijms24010854

**Published:** 2023-01-03

**Authors:** Natalie Oberhuber, Hindole Ghosh, Bianca Nitzsche, Prasad Dandawate, Michael Höpfner, Rainer Schobert, Bernhard Biersack

**Affiliations:** 1 Organic Chemistry 1, University of Bayreuth, Universitätsstrasse 30, 95440 Bayreuth, Germany; 2 Department of Cancer Biology, University of Kansas Medical Center, 3901 Rainbow Boulevard, Kansas City, KS 66160, USA; 3 Institute of Physiology, Charité-Universitätsmedizin Berlin, Corporate Member of Freie Universität Berlin, Humboldt-Universität zu Berlin and Berlin Institute of Health, Charitéplatz 1, 10117 Berlin, Germany

**Keywords:** anticancer agents, kinase inhibitors, tyrphostin, indoles, VEGFR inhibition, molecular docking

## Abstract

New *N*-alkylindole-substituted 2-(pyrid-3-yl)-acrylonitriles with putative kinase inhibitory activity and their (*p*-cymene)Ru(II) piano-stool complexes were prepared and tested for their antiproliferative efficacy in various cancer models. Some of the indole-based derivatives inhibited tumor cell proliferation at (sub-)micromolar concentrations with IC_50_ values below those of the clinically relevant multikinase inhibitors gefitinib and sorafenib, which served as positive controls. A focus was set on the investigation of drug mechanisms in HCT-116 p53-knockout colon cancer cells in order to evaluate the dependence of the test compounds on p53. Colony formation assays as well as experiments with tumor spheroids confirmed the excellent antineoplastic efficacy of the new derivatives. Their mode of action included an induction of apoptotic caspase-3/7 activity and ROS formation, as well as anti-angiogenic properties. Docking calculations with EGFR and VEGFR-2 identified the two 3-aryl-2-(pyrid-3-yl)acrylonitrile derivatives **2a** and **2b** as potential kinase inhibitors with a preferential activity against the VEGFR-2 tyrosine kinase. Forthcoming studies will further unveil the underlying mode of action of the promising new derivatives as well as their suitability as an urgently needed novel approach in cancer treatment.

## 1. Introduction

**Figure 1:**
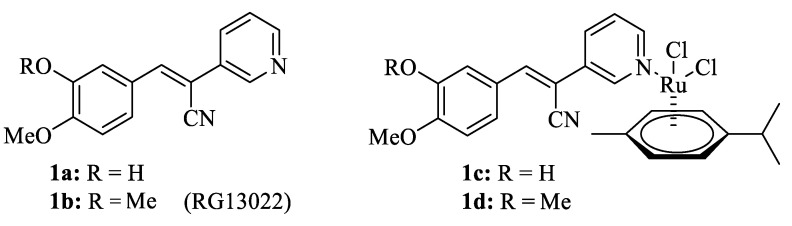
Structures of the anticancer active tyrphostins **1a**–**d**.

Heterocycles are often used for the design of new and potent drug candidates [1]. In addition, the Knoevenagel reaction is a straightforward method to form C=C bonds, and the Knoevenagel reaction from aryl acetonitriles and aryl aldehydes was applied for the preparation of aryl-substituted acrylonitriles [2,3]. Analogous tyrphostins (tyrosine kinase phosphorylation inhibitors) are promising anticancer compounds because they can inhibit vital growth factor receptor tyrosine kinases such as the epidermal growth factor receptor (EGFR) [4]. Gefitinib, erlotinib, and afatinib are clinically approved EGFR inhibitors with activity against EGFR-positive lung adenocarcinomas [5]. Several antitumor active derivatives with aminophenyl, halophenyl or heterocyclic moieties, e.g., the pyridine derivatives **1a** and **1b** (RG13022), were reported [6,7,8,9,10,11,12,13]. Pyridine-based compounds are excellent ligands for various metal complexes, and the (arene)Ru(II) complexes **1c** and **1d** of the tyrphostins **1a** and **1b** showed increased selectivity and antiproliferative activity against tumor cells (Figure 1) [14]. Ruthenium complexes are attractive anticancer drug candidates [15]. The classical “iconic” Ru(III) complexes NAMI-A and KP1019 underwent clinical trials [15,16]. While NAMI-A has a pronounced anti-metastatic activity combined with low cytotoxicity, KP1019 is able to exert distinct cytotoxic effects on tumor cells [17]. However, there are further mechanisms of action for these Ru(III) complexes [18]. In terms of non-classical piano-stool (arene)Ru(II) complexes, noteworthy drug candidates are anti-metastatic complexes with pta (1,3,5-triaza-7-phosphaadamantane) ligands (i.e., RAPTA complexes), as well as various cytotoxic [(arene)Ru(en)Cl]^+^ complexes, which were able to overcome cisplatin-resistance in cancer cells [19,20]. Inspired by these Ru(II) complexes, further anticancer active (arene)Ru(II) complexes were described as inhibitors of glutathione-S-transferase and of the drug efflux transporter P-glycoprotein [21,22]. The identification of anticancer active (arene)Ru(II) *N*-heterocyclic carbene complexes is another remarkable development [23,24].

In this work, we present new 3-pyridyl-substituted indole-based acrylonitriles and their (*p*-cymene)dichloridoruthenium(II) complexes as highly active compounds against tumor cells. The applied indole scaffolds replaced the isovanillyl and veratryl moieties of the known compounds **1a**–**d** with the intention to enhance anticancer activity. Their effects on antiproliferative activity, apoptosis induction, and protein kinase inhibition were studied by biological and bioinformatic evaluations, giving a first insight into their antineoplastic efficacy and mode of action.

## 2. Results

### 2.1. Chemistry

**Scheme 1:**
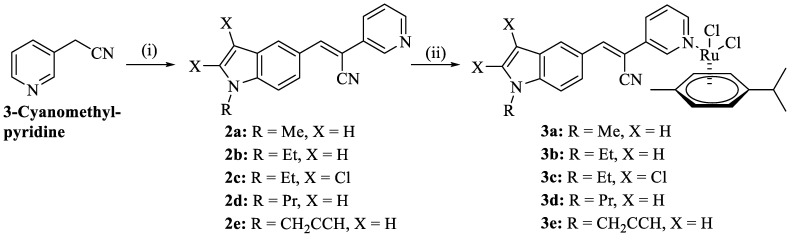
Reagents and conditions: (i) Aryl aldehyde, piperidine (cat.), EtOH, reflux, 24 h, 35–67%; (ii) [Ru(*p*-cymene)Cl_2_]_2_, CH_2_Cl_2_, r.t., 3 h, 61–82%.

The synthesis of the new indole-based tyrphostins **2a**–**e** and their ruthenium complexes **3a**–**e** was carried out following procedures in the literature [13,14]. The Knoevenagel reaction of 3-cyanomethylpyridine with substituted aryl aldehydes in hot ethanol in the presence of a cat. amount of piperidine. The new dichlorido(*p*-cymene)ruthenium(II) complexes were prepared from the pyridine-based tyrphostins as described previously (Scheme 1) [14].

### 2.2. Anticancer Activity

Initially, the antiproliferative activities of ten new compounds named **2a**–**e** and **3a**–**e** against HCT-116 colorectal carcinoma (both p53-wildtype and p53-knockout cells) and MCF-7 breast carcinoma cells were evaluated using the MTT assay (Table 1). The known kinase inhibitors sorafenib and gefitinib, as well as the known anti-metastatic ruthenium complex NAMI-A were used as control compounds. Among compounds **2a**–**e**, derivative **2b** exhibited the highest activities against both colorectal cancer cell lines, while **2a** was the most active analog against MCF-7 cells. Both **2a** and **2b** were distinctly more active than sorafenib and gefitinib. While **2b** revealed high activities with IC_50_ values in the sub-micromolar concentration range against both HCT-116 cell lines, **2a** was distinctly more active against the p53-knockout cells than against the HCT-116 p53-wildtype cells. Compound **2c** showed lower activities than **2a** and **2b**, in the activity range of the positive control sorafenib. **2d** and **2e** were inactive in the applied cancer cell lines. Significant results were also obtained for the ruthenium complexes **3a** and **3b**, which contain **2a** and **2b** as ligands. Complex **3a** was especially active against HCT-116 wildtype cells, and against MCF-7 cells. In these cells, **3a** showed higher activities than its ligand **2a**, while **2a** was more active than **3a** against the HCT-116 p53-negative cells. **3a** was also much more active against all three cell lines than the positive controls sorafenib, gefitinib, and NAMI-A. Complex **3b** conserved the activity of its ligand **2b** against the MCF-7 cells but was 3- to 10-fold less active than **2b** against both HCT-116 cell lines. Complex **3c** was more active than **2c** against MCF-7 cells but showed no activity against cells of both HCT-116 cell lines. Complexes **3d** and **3e** were as inactive as their ligands **2d** and **2e**.

The most active compounds **2a**, **2b** and **3a** were selected and tested for their activity against further cancer cell lines such as topotecan-resistant MCF-7/Topo, 518A2 melanoma, EaHy.926 hybrid endothelial and lung carcinoma, and Huh-7 hepatocellular carcinoma (Table 2). Complex **3a** and its ligand **2a** were especially active against multi-drug resistant MCF-7/Topo breast carcinoma cells (IC_50_ values of 0.18 μM and 0.10 μM, respectively), and distinctly more active than against the non-resistant MCF-7 cells (Table 1). **3a** was also very active against 518A2 melanoma cells (IC_50_ = 0.6 μM), and more active than its ligand **2a** (IC_50_ = 1.4 μM). Both **2a** and **3a** were more active than **2b** (IC_50_ = 1.5 μM for MCF-7/Topo and 2.8 μM for 518A2) and the positive controls sorafenib and gefitinib against 518A2 and MCF-7/Topo cells. In the endothelial cell line EaHy.926, which is a hybrid of endothelial cells and lung cancer cells, compounds **2a** and **2b** (IC_50_ = 0.9–1.3 μM) were slightly more active than complex **3a** (IC_50_ = 1.9 μM), yet all three compounds were distinctly more active than sorafenib and gefitinib. However, compounds **2a** and **3a** were most active against Huh-7 hepatocellular carcinoma cells with excellent IC_50_ values of 0.01–0.04 μM. Gefitinib was inactive against Huh-7 cells, while sorafenib also showed an amenable activity against these tumor cells (IC_50_ = 0.9 μM), albeit distinctly less than **2a** and **3a**. Finally, the compounds **2a**, **2b** and **3a** were tested in two esophageal adenocarcinoma cell lines (FLO-1 and SK-GT4) using the hexosaminidase assay. Compound **2a** exhibited considerable activity against FLO-1 cells (IC_50_ = 1.2 μM), while it was inactive against SK-GT-4 cells. **2b** was inactive against both esophageal cancer cell lines. Complex **3a** was slightly less active against FLO-1 cells (IC_50_ = 2.5 μM) than its ligand **2a**, yet it was likewise inactive against SK-GT-4 cells.

**Figure 2:**
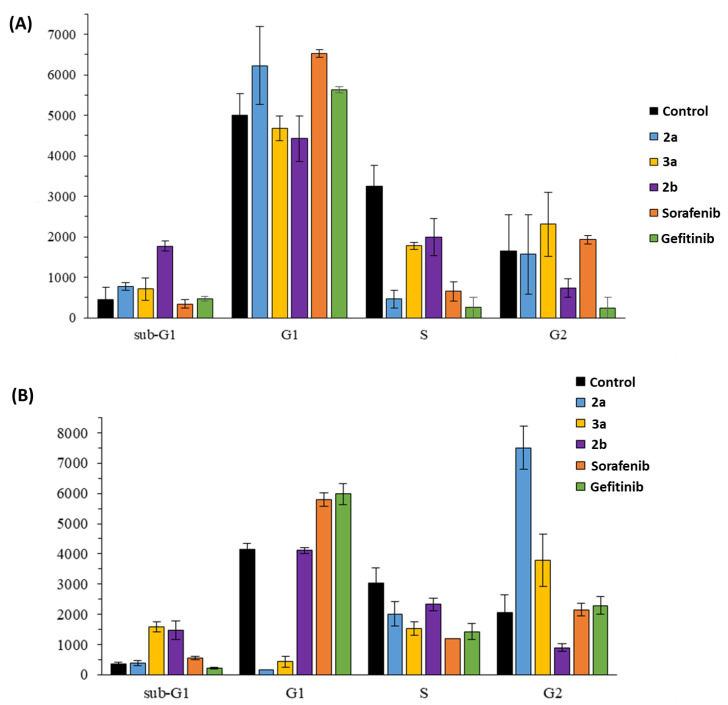
Effects of **2a**, **2b** and **3a** (IC_50_ concentrations) on the cell cycle of HCT-116 wildtype (**A**); and p53-knockout cells (**B**) after 24 h. Sorafenib (IC_50_) and gefitinib (50 μM for HCT-116 wildtype and IC_50_ for p53-knockout cells) were used as positive controls. y-axis: number of cells. n = 2 ± SD.

Based on the promising antiproliferative activities of **2a**, **2b** and **3a**, mechanistic studies of these compounds with HCT-116 wildtype and HCT-116 p53-knockout cells were carried out in order to discover hints at any dependencies on p53. Initially, cell cycle analyses of **2a**, **2b** and **3a** were carried out (Figure 2). Compound **2a** led to a G1 phase arrest in HCT-116 wildtype cells, which is in line with the effects of sorafenib and gefitinib on the cell cycle of these cells, while compound **2b** showed no cell cycle arrest but led to increased sub-G1 numbers indicating enhanced cell death. In contrast to that, complex **3a** induced to a G2 phase arrest in the HCT-116 wildtype cells. In the p53-knockout cells, both **2a** and **3a** led to distinct G2 phase arrest, complex **3a** also showed an increase in sub-G1 cell percentage indicating cell death. Compound **2b** showed increased sub-G1 cell level and no effects on the cell cycle again. Sorafenib and gefitinib exhibited G1 phase arrest in the knockout cells, too.

**Figure 3:**
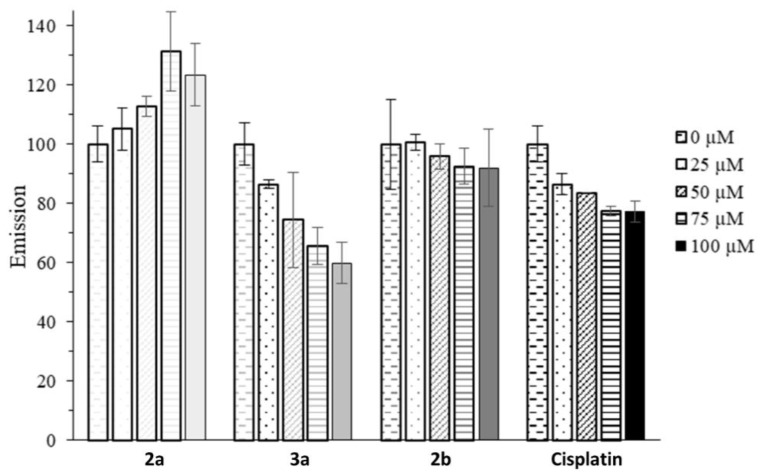
DNA binding of **2a**, **2b** and **3a** using the ethidium bromide intercalation assay with salmon NA. Cisplatin was used as positive control. n = 4 ± SD.

DNA binding is a possible mode of action of acrylonitriles and dichloridoruthenium(II) complexes. The cell-free ethidium bromide intercalation assay was applied in order to identify (salmon) DNA binding by compounds **2a**, **2b** and **3a**. While **2a** and **2b** exhibited no DNA binding, the ruthenium complex **3a** showed a strong DNA interaction, which was stronger than the DNA binding by the known DNA-damaging anticancer drug cisplatin (Figure 3).

**Figure 4:**
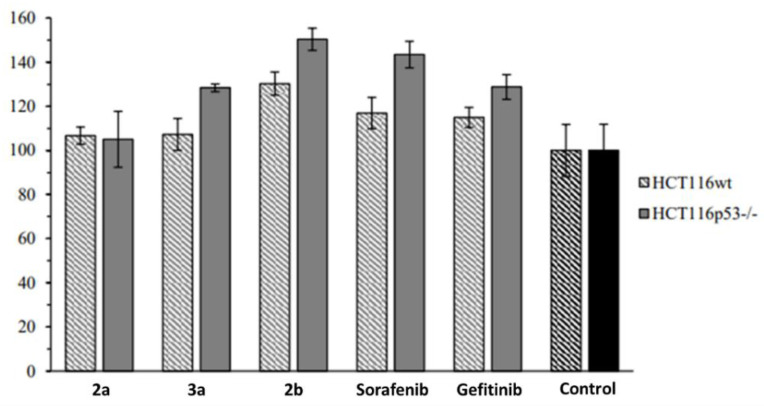
ROS formation by **2a**, **2b** and **3a** (4 × IC_50_) in HCT-116 wildtype and p53-knockout cells after 24 h. Sorafenib (4 × IC_50_) and gefitinib (200 μM for HCT-116 wildtype cells and 4 × IC_50_ for p53-knockout cells) were used as positive controls. y-axis: Fluorescence in % with control set to 100%. n = 3 ± SD.

Next, the formation of reactive oxygen species (ROS) by **2a**, **2b** and **3a** in p53 wildtype and p53-knockout HCT-16 cells were studied. Compound **2b** and complex **3a** showed distinctly increased ROS levels in the p53-knockout cells when compared with the wildtype cells and the ROS formation by **2a**. Sorafenib and gefitinib also displayed higher levels of ROS in the p53-knockout cells (Figure 4).

**Figure 5:**
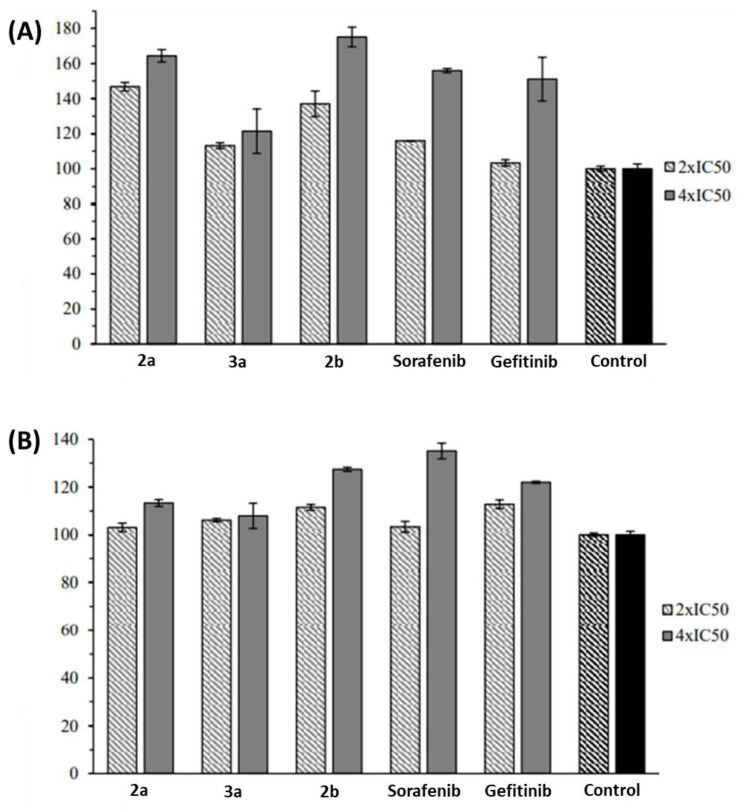
Caspase 3/7 activation by **2a**, **2b** and **3a** in HCT-116 wildtype (**A**) and p53-knockout cells (**B**) after 24 h. Sorafenib and gefitinib (100 μM and 200 μM in wildtype cells) were used as positive controls. y-axis: caspase-3/7 activity in % with control set to 100%. n = 3 ± SD.

**Figure 6:**
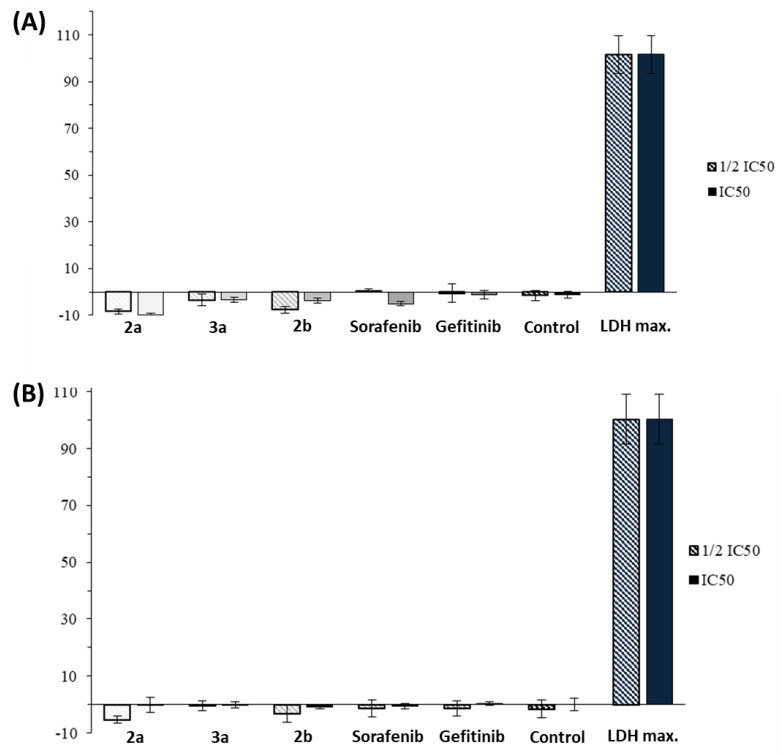
LDH release by **2a**, **2b** and **3a** in HCT-116 wildtype (**A**); and p53-knockout (**B**) cells. Sorafenib and gefitinib (25 μM and 50 μM for HCT-116 wildtype cells) were used as positive controls. y-axis: LDH release in %. n = 3 ± SD.

Drug-induced apoptosis by activation of effector caspases 3/7 in p53-wildtype and p53-knockout HCT-116 cells was investigated (Figure 5). Induction of apoptosis by **2a**, **2b** and **3a** was dose-dependent in the HCT-116 wildtype cells. When compared with the negative control, compound **2a** increased caspase activation by more than 60% (at 4 × IC_50_). Compound **2b** enhanced caspases activation by 37% (2 × IC_50_) and 75% (4 × IC_50_). Complex **3a** showed the weakest caspase activity increase. Weaker apoptosis induction activities of **2a**, **2b** and **3a** were observed in the p53-knockout cells. Complex **3a** only led to a minimal increase, compound **2a** led to an increase by 13%, and **2b** led to an increase by 27% at high doses (4 × IC_50_). The unspecific toxicity of compounds **2a**, **2b** and **3a** was tested using the LDH (lactate dehydrogenase) assay. No LDH release was observed for the test compounds in both HCT-116 cell lines (Figure 6). Thus, an induction of necrotic cell death by the test compounds can be excluded.

**Figure 7:**
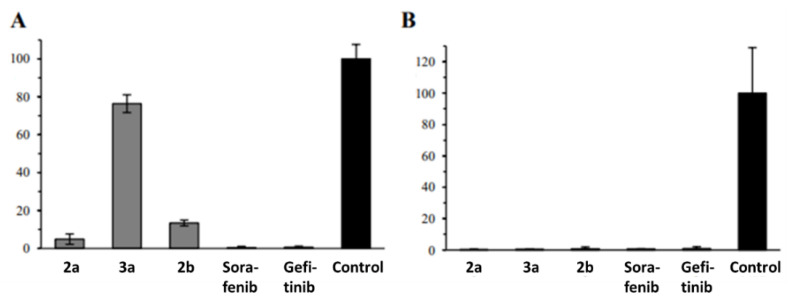
Inhibition of colony formation by **2a**, **2b** and **3a** at their IC_50_ doses in HCT-116 wildtype (**A**); and HCT-116 p53-knockout cells (**B**). Sorafenib (IC_50_) and gefitinib (20 μM for wildtype, IC_50_ for p53-knockout cells) were used as positive controls. y-axis: Percentage of colonies compared to control, which was set to 100%. n = 3 ± SD.

**Figure 8:**
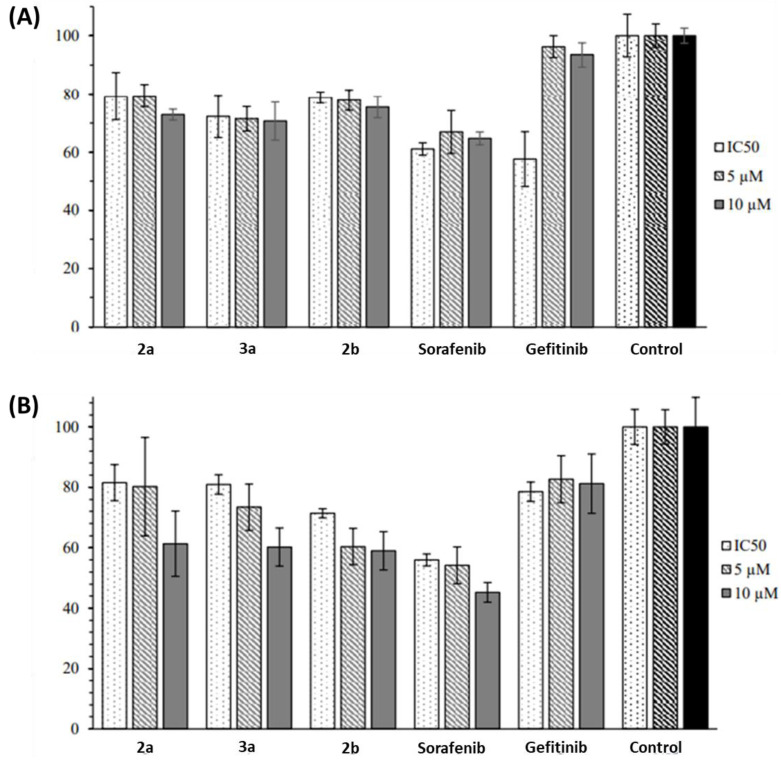
Suppression of tumor spheroids by **2a**, **2b** and **3a** in HCT-116 wildtype cells (**A**); and HCT-116 p53-knockout cells (**B**). Sorafenib and gefitinib (50 μM in the wildtype cells) were used as positive controls. y-axis: spheroid diameter in % with control set to 100%. n = 3 ± SD.

**Figure 9:**
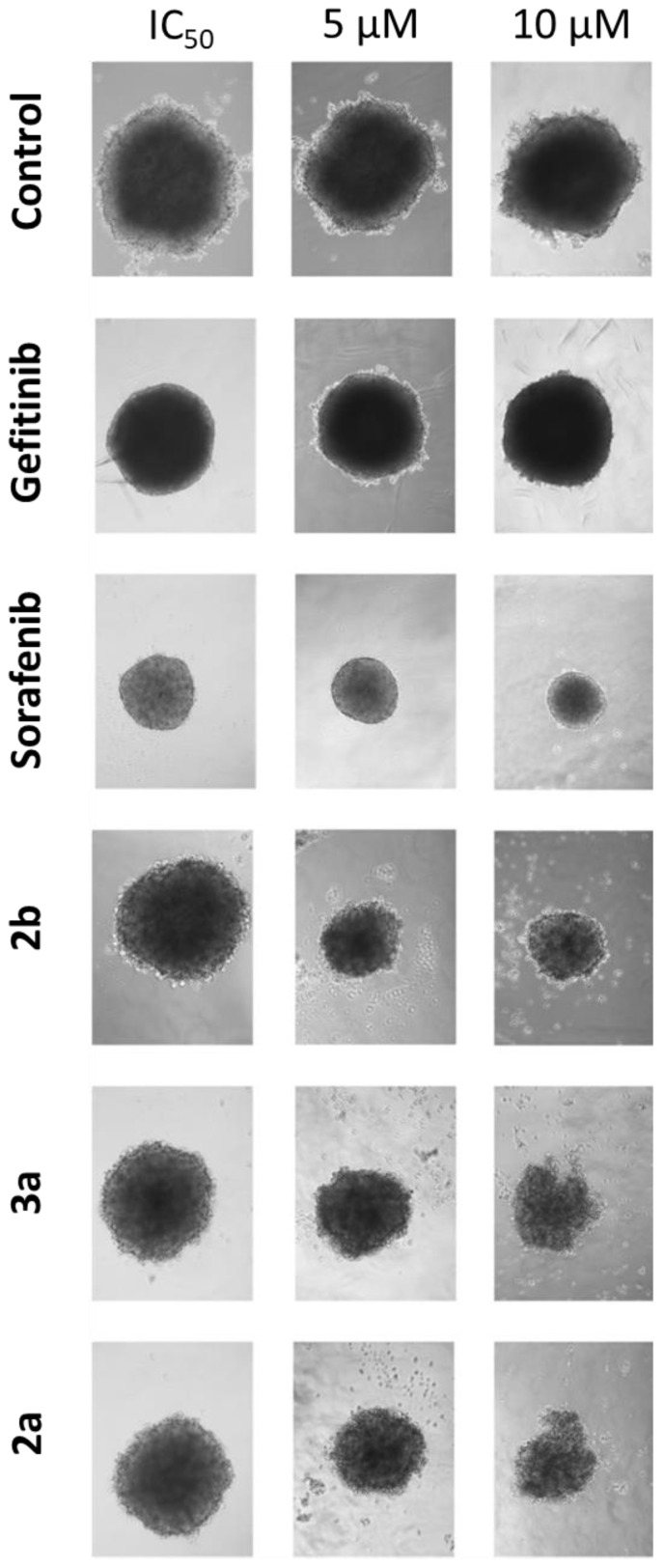
Suppression of tumor spheroids by **2a**, **2b** and **3a** in HCT-116 p53-knockout cells. Sorafenib and gefitinib were used as positive controls. Representative images from four experiments, 100× magnification.

Compounds **2a**, **2b** and **3a** were also tested for their ability to suppress colony formation by colon cancer cells (Figure 7). In the HCT-116 wildtype cells, **2a** and **2b** considerably inhibited the formation of tumor cell colonies, albeit a little less pronounced than sorafenib and gefitinib. Complex **3a** showed only weak inhibitory activity in these cells. In contrast to that, all three test compounds **2a**, **2b** and **3a** strongly inhibited the colony formation by p53-knockout cells, and virtually no colonies were detected upon treatment with these compounds. Next, the inhibition of tumor spheroids by the test compounds **2a**, **2b** and **3a** was investigated (Figure 8 and Figure 9). All three test compounds inhibited HCT-116 wildtype spheroid formation (reduction of spheroid diameter by 20–30%) with the best performance being achieved complex **3a** performing. Only sorafenib was slightly more efficient at suppressing these spheroids (reduction of spheroid diameter of up to 40%). Again, stronger suppressing effects were observed in the spheroids formed by p53-knockout cells treated with **2a**, **2b** and **3a**. At higher doses, these compounds led to spheroids, which showed only 60% of the diameter of untreated spheroids. Yet again, sorafenib performed best in these cells in terms of spheroid inhibition.

**Figure 10:**
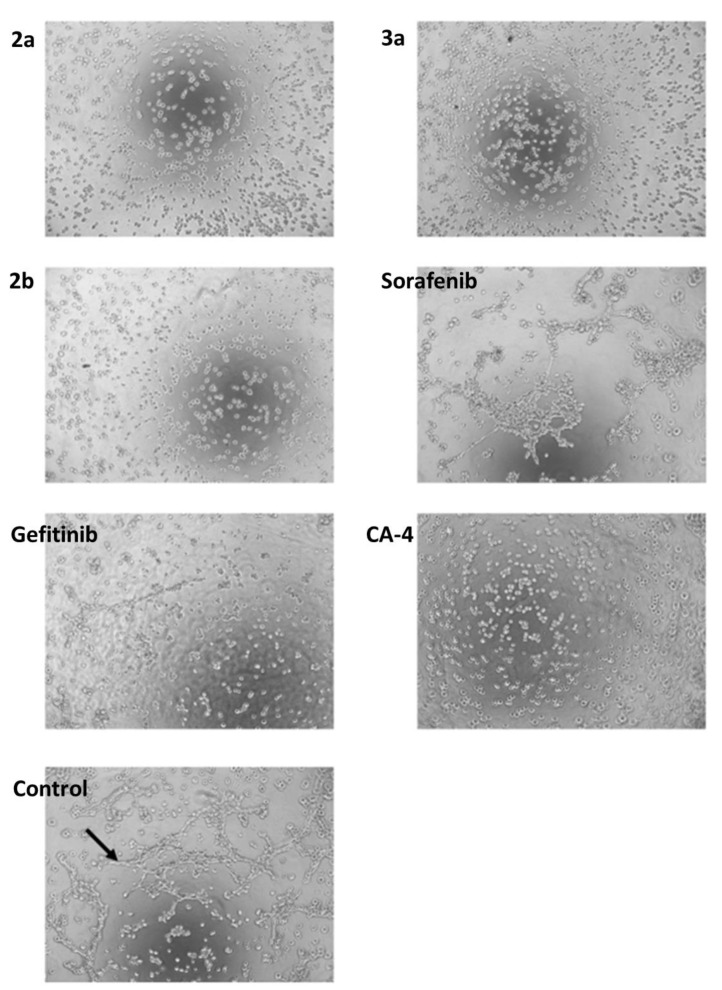
Suppression of tube formation by EaHy.926 cells upon treatment with **2a**, **2b** and **3a** (5 μM) for 6 h. Sorafenib (5 μM), gefitinib (5 μM), and combretastatin A-4 (CA-4, 10 nM) were used as positive controls.

Together with the assumption, that **2a**, **2b** and **3a** might be VEGFR-2 inhibitors similar to previously published thienyl analogs, these promising results from tests with HCT-116 colorectal cancer cells prompted us to study the anti-angiogenic activity of compounds **2a**, **2b** and **3a** as a possible mode of action [13]. For this reason, the tube formation assay using the hybrid EAHy.926 cells, which are able to form endothelial tubes, was applied. All three test compounds inhibited tube formation efficiently, and only round cells were visible upon treatment with these compounds. In contrast, proper tubes were still observed in the experiments using sorafenib and gefitinib indicating a lower antiangiogenic activity of these approved kinase inhibitors when compared with the activities of **2a**, **2b** and **3b** (Figure 10). As expected, the highly vascular disrupting positive control combretastatin A-4 also blocked tube formation at low doses.

**Figure 11:**
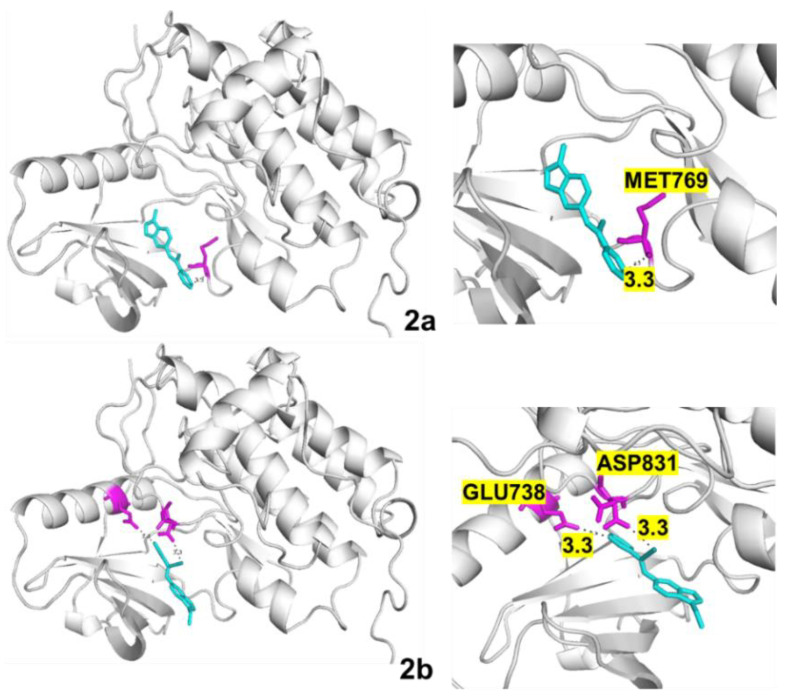
Molecular docking of **2a** and **2b** into EGFR.

**Figure 12:**
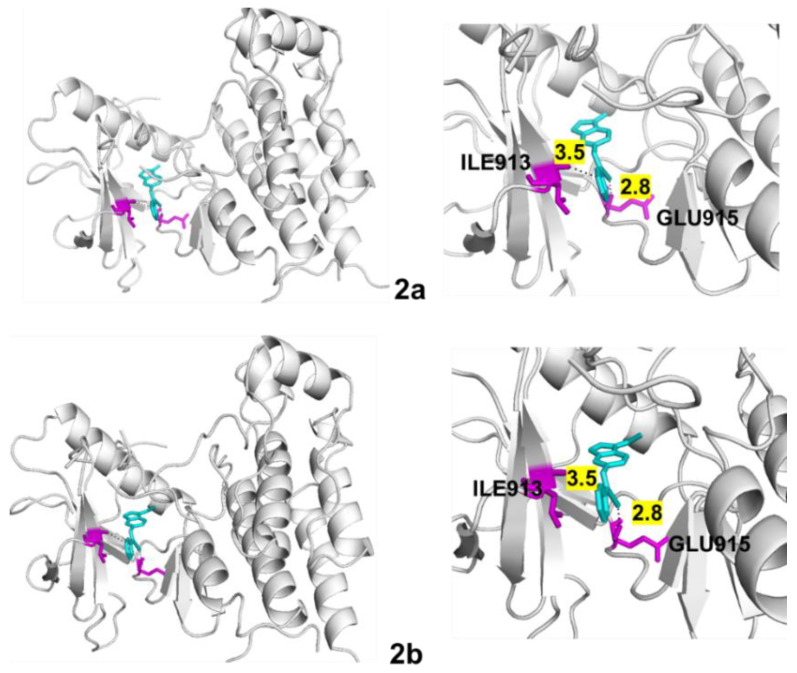
Molecular docking of **2a** and **2b** into VEGFR-2.

Finally, molecular docking of **2a** and **2b** into the protein structures of EGFR and VEGFR-2 was performed using Autodock vina. **2a** and **2b** bound to EGFR with favorable binding energies of −8.2 and −7.7 kcal/mol, respectively (Table 3). However, their binding modes differed and included H bonds with GLU738 and ASP831 for **2b**, while **2a** formed only one H bond with MET769 (Table 3, Figure 11). More favorable affinities (−9.5 kcal/mol) and identical binding modes were determined for both **2a** and **2b** when docked into VEGFR-2 (Table 3, Figure 12). **2a** and **2b** stabilized themselves in the protein cavity of VEGFR-2 by forming two H bonds with the amino acids ILE913 and GLU915.

## 3. Discussion

The synthesis of the new indole-based compounds **2a**–**e** and their (*p*-cymene)dichloridoruthenium(II) complexes **3a**–**e** is straightforward. Initial antiproliferative tests against three solid tumor cell lines revealed promising activities of the indoles **2a** and **2b**, as well as of the Ru complexes **3a** and **3b**. *N*-methyl and *N*-ethylindole derivatives were much more active than *N*-propyl and *N*-propargylindole analogs, which were inactive. Thus, only short *N*-alkyl substituents (C_1_ and C_2_) exhibited antiproliferative activities, and already C3 substituents of the indole nitrogen (propyl and propargyl) had strongly inactivating effects. A proper indole scaffold is necessary for a high antiproliferative activity since 2,3-dichloroindoles **2c** and **3c** were distinctly less active than **2a**–**b** and **3a**–**b**, except for the MCF-7 cells, where complex **3c** was only slightly less active than complex **3b**. When compared with their metal-free ligands **2**, only complexes **3a** and **3c** surpassed the activity of their ligands, namely **2a** (in HCT-116 wildtype, MCF-7, MCF-7/Topo, 518A2, and Huh-7 cells) and **2c** (in MCF-7 cells). In the case of **2b**, ruthenation led to a reduction of antiproliferative activity. Interestingly, compounds **2a**, **2b** and **3a** distinctly surpassed the antiproliferative activity of the approved kinase inhibitors sorafenib and gefitinib in all the used cancer cell lines. Complex **3a** overcame topotecan-resistance of MCF-7/Topo cells associated with BCRP overexpression (BCRP = breast cancer resistance protein, an ABC drug efflux transporter also known as ABCG2), while **2a** showed a remarkable preference for p53-knockout cells when compared with the corresponding HCT-116 wildtype cells. Topotecan is a promising drug for the treatment of advanced breast cancer, and complex **3a** might be tested accordingly in combination with topotecan and other anticancer drug substrates of BCRP in future pre-clinical studies [25,26]. Several BCRP and dual P-gp/BCRP inhibitors were described as sensitizers to anticancer drug treatment in BCRP-overexpressing cancer cells [27]. A reversal of BCRP-induced multidrug resistance was already reported for certain kinase inhibitors, such as the VEGFR-2 inhibitor ZM323881 and regorafenib, a clinically approved multi-kinase inhibitor (inhibitor of VEGFR2-TIE2, VEGFR1/3, FGFR-1 and PDGFR-β) [28,29]. It is noteworthy that the known isovanillyl analogs **1a** and **1c** have also shown relatively high antiproliferative activities against MCF-7/Topo cells, indicating a considerable sensitivity of these multidrug-resistant cells for these compounds [14].

The high activities of **2a** and **2b** against HCT-116 p53-knockout cells is remarkable in the light of a growing number of promising MDM2-inhibitory imidazolines, i.e., the “nutlins”, and of indoles, which activate p53 by blocking its interaction with its negative regulator MDM2 to enforce apoptosis in cancer cells [30,31]. MDM2 promotes the ubiquitination and proteasomal degradation of p53 in colon cancer cells, and quite a few MDM2 inhibitors have entered clinical trials for the treatment of various cancers [32]. However, the formation of nutlin-resistance poses a considerable problem for the therapeutic outcome of p53 activators, which might be solved by p53-independent apoptosis inducers such as **2a** and **2b** [33]. In contrast to that, the especially high activity of the dichloridoruthenium(II) complex **3a** against p53-wildtype HCT-116 might be based on its DNA-binding properties analogously to the p53-dependent DNA-damaging mechanism of approved platinum-based drugs. The metal-free compounds **2a** and **2b** lack any DNA-binding activity.

The preliminary mechanistic investigation of compounds **2a**, **2b** and **3a** in HCT-116 wildtype and p53-knockout cells elucidated remarkable properties of these compounds. ROS formation was higher in treated p53-knockout cells than in wildtype cells. The suppression of colony formation and tumor spheroids by the test compounds was more pronounced in the p53-knockout cell line. In addition, anti-angiogenic activities were observed for **2a**, **2b** and **3a** in tube formation experiments using EaHy.926 cells, which is in line with docking calculations, indicating high affinities of **2a** and **2b** for VEGFR-2 (the metal complex **3a** could not be docked by the described methods). Higher inhibitory activities against VEGFR-2 were described recently for a close thienyl analog of **2a** when compared with other kinases [34]. Interestingly, there exists a connection between p53-deficiency and increased angiogenesis and colon cancer growth via cancer-associated fibroblasts [35]. The TP53 mutation state predicts the response of colon cancer patients to VEGFR-2 inhibitor treatment, and patients with p53-mutant cancers revealed improved responses to inhibitor treatment, probably by activation of T-cell response [36]. It remains to be shown if **2a**, **2b** and **3a** can cause similar beneficial effects in future in vivo studies, but the obtained data from the treatment of p53-knockout cells appears promising in this regard.

While no necrotic effects were observed for **2a**, **2b** and **3a**, apoptosis induction by **2a** and **2b** was more pronounced in the HCT-116 wildtype cells than in the p53-knockout cells. This indicates a preference of these compounds for the p53-dependent intrinsic apoptosis pathway. Apoptosis induced by complex **3a** was lower in both cell lines, and seemingly less dependent on p53. The induction of apoptosis in the p53-knockout cells at higher doses of the test compounds might occur via the extrinsic TRAIL-induced apoptosis pathway, as it was shown previously for gefitinib [37,38]. In addition, other cell death mechanisms can be assumed for compounds **2b** and **3a** when considering their relatively high sub-G1 phase cell numbers in the p53-knockout cells. It is remarkable that **2a** showed G1 phase arrest in the wildtype HCT-116 cells, and G2 phase arrest in the p53-knockout cells. In contrast, gefitinib and sorafenib only led to G1 phase arrest in both cell lines. Thus, protein kinase-independent mechanisms might play a role for the **2a**-induced G2 phase arrest in the HCT-116 p53-knockout cells. Various p53-independent signaling pathways with relevance for G2/M phase transition in cancer cells were described [39]. In addition, p53-independent induction of G2/M arrest in colon cancer cells was described for diallyl disulfide, a major component of garlic oil. After knockdown of p53 by siRNA in HCT-116 cells, diallyl disulfide treatment still led to G2/M arrest accompanied by increased cyclin B1 expression [40].

Considering the antiproliferative activities of **2a** and **3a** against all tested cell lines. Huh-7 hepatocellular carcinoma cells turned out to be especially sensitive to **2a** and **3a**, with IC_50_ values in the low nanomolar range. Huh-7 cells express a mutant p53 form, which is more stable than wildtype p53 but transcriptionally inactive [41,42,43]. Thus, further tests of the anticancer active indole-based compounds using various liver tumor cell lines with aberrant p53 appear to be very promising in the light of the described results from the couple of HCT-116 p53-wildtype and knockout colon cancer cell lines.

## 4. Materials and Methods

### 4.1. General Procedures

Column chromatography: silica gel 60 (230–400 mesh). Melting points (uncorrected), Electrothermal 9100; IR spectra, Perkin-Elmer Spectrum One FT-IR spectrophotometer with ATR sampling unit; NMR spectra, Bruker Avance 300 spectrometer; chemical shifts are given in parts per million (*δ*) downfield from tetramethylsilane as internal standard; Mass spectra, Thermo Finnigan MAT 8500 (EI), UPLC/Orbitrap (ESI-HRMS).

### 4.2. Materials

Starting compounds and reagents were obtained from abcr (Karlsruhe, Germany), Sigma-Aldrich (Darmstadt, Germany) and TCI (Zwijndrecht, Belgium). The alkylated 5-formylindole precursors were prepared according to literature procedures, and the obtained analytical data for these indole derivatives was in line with published data [44,45].

### 4.3. Synthesis

#### 4.3.1. Synthesis of Acrylonitriles **2**

##### 3-[1-Cyano-2-(1-methylindol-5-yl)-(*Z*)-ethenyl)]-3-pyridine (**2a**)

*N*-Methyl-indol-5-carboxaldehyde (159 mg, 1.0 mmol) was dissolved in ethanol (p.a., 5 mL) and 3-pyridineacetonitrile (118 mg, 1.0 mmol) was added. A catalytic amount of piperidine (eight drops via a Pasteur pipette) was added and the reaction mixture was vigorously stirred under reflux for 24 h. After cooling to room temperature, the formed precipitate was collected, washed with EtOH/water and dried in vacuum. Yield: 122 mg (0.47 mmol, 47%); yellow solid of mp 133–135 °C; *υ*_max_(ATR)/cm^−1^ 3100, 3032, 2945, 2919, 2827, 2207, 1591, 1566, 1508, 1487, 1453, 1428, 1415, 1387, 1362, 1329, 1309, 1249, 1230, 1169, 1160, 1134, 1105, 1079, 1025, 991, 924, 907, 881, 851, 825, 802, 781, 757, 720, 699, 662; ^1^H NMR (300 MHz, DMSO-d_6_) *δ* 3.85 (3 H, s), 6.60 (1 H, s), 7.4–7.5 (2 H, m), 7.62 (1 H, d, J = 8.7 Hz), 7.88 (1 H, d, J = 8.7 Hz), 8.1–8.2 (1 H, m), 8.19 (1 H, s), 8.24 (1 H, s), 8.6–8.7 (1 H, m), 8.96 (1 H, s); ^13^C NMR (75.5 MHz, CDCl_3_) *δ* 32.7, 101.8, 102.7, 110.4, 118.3, 122.3, 123.8, 124.7, 128.1, 130.7, 131.5, 132.8, 137.6, 146.3, 146.5, 149.2; *m*/*z* (%) 259 (100) [M^+^], 243 (24), 216 (13).

##### 3-[1-Cyano-2-(1-ethylindol-5-yl)-(*Z*)-ethenyl)]-3-pyridine (**2b**)

1-Ethylindol-5-carboxaldehyde (173 mg, 1.0 mmol) was dissolved in ethanol (p.a., 5 mL) and 3-pyridineacetonitrile (118 mg, 1.0 mmol) was added. A catalytic amount of piperidine (eight drops via a Pasteur pipette) was added and the reaction mixture was vigorously stirred under reflux for 24 h. After cooling to room temperature, the solvent was evaporated, and the residue was recrystallized from CH_2_Cl_2_/*n*-hexane. Yield: 125 mg (0.46 mmol, 46%); yellow solid of mp 92–93 °C; *υ*_max_(ATR)/cm^−1^ 3136, 2983, 2943, 2890, 2206, 1598, 1579, 1568, 1490, 1466, 1427, 1402, 1358, 1325, 1302, 1283, 1264, 1228, 1165, 1145, 1133, 1088, 1052, 1022, 992, 964, 940, 918, 867, 828, 814, 789, 756, 715, 706, 662; ^1^H NMR (300 MHz, DMSO-d_6_) *δ* 1.38 (3 H, t, J = 7.2 Hz), 4.26 (2 H, q, J = 7.2 Hz), 6.5–6.6 (1 H, m), 7.5–7.6 (2 H, m), 7.66 (1 H, d, J = 8.7 Hz), 7.87 (1 H, d, J = 8.7 Hz), 8.1–8.2 (1 H, m), 8.20 (1 H, s), 8.24 (1 H, s), 8.5–8.6 (1 H, m), 8.97 (1 H, s); ^13^C NMR (75.5 MHz, DMSO-d_6_) *δ* 15.5, 102.0, 102.6, 110.4, 118.3, 122.2, 123.5, 123.9, 124.7, 128.2, 130.0, 130.6, 132.8, 136.7, 146.3, 146.5, 149.2; HRMS for C_18_H_16_N_3_ [M^+^ + H] calcd. 274.13387, found 274.13367.

##### 3-[1-Cyano-2-(2,3-dichloro-1-ethyl-indol-5-yl)-(*Z*)-ethenyl)]-3-pyridine (**2c**)

2,3-Dichloro-1-ethyl-indol-5-carboxaldehyde (242 mg, 1.0 mmol) was dissolved in ethanol (p.a., 5 mL) and 3-pyridineacetonitrile (118 mg, 1.0 mmol) was added. A catalytic amount of piperidine (eight drops via a Pasteur pipette) was added and the reaction mixture was vigorously stirred under reflux for 24 h. After cooling to room temperature, the formed precipitate was collected, washed with EtOH/water and dried in vacuum. Yield: 120 mg (0.35 mmol, 35%); yellow solid of mp 180 °C; *υ*_max_(ATR)/cm^−1^ 3045, 2972, 2935, 2874, 2211, 1616, 1600, 1583, 1569, 1522, 1486, 1475, 1464, 1450, 1431, 1394, 1366, 1347, 1335, 1312, 1279, 1259, 1227, 1186, 1161, 1124, 1113, 1081, 1056, 1023, 995, 945, 879, 828, 803, 797, 774, 746, 699, 985; ^1^H NMR (300 MHz, DMSO-d_6_) *δ* 1.30 (3 H, t, J = 7.0 Hz), 4.35 (2 H, q, J = 7.0 Hz), 7.5–7.6 (1 H, m), 7.82 (1 H, d, J = 8.7 Hz), 7.97 (1 H, d, J = 8.7 Hz), 8.1–8.2 (2 H, m), 8.28 (1 H, s), 8.6–8.7 (1 H, m), 8.97 (1 H, s); ^13^C NMR (75.5 MHz, DMSO-d_6_) *δ* 14.8, 101.9, 104.7, 111.3, 117.9, 119.4, 123.3, 123.8, 123.9, 126.5, 130.2, 133.0, 134.3, 145.1, 146.6, 149.6; HRMS for C_18_H_14_N_3_Cl_2_ [M^+^ + H] calcd. 342.05593, found 342.05567.

##### 3-[1-Cyano-2-(1-propylindol-5-yl)-(*Z*)-ethenyl)]-3-pyridine (**2d**)

1-Propylindol-5-carboxaldehyde (332 mg, 1.8 mmol) was dissolved in ethanol (p.a., 5 mL) and 3-pyridineacetonitrile (210 mg, 1.8 mmol) was added. A catalytic amount of piperidine (eight drops via a Pasteur pipette) was added and the reaction mixture was vigorously stirred under reflux for 24 h. After cooling to room temperature, the formed precipitate was collected, washed with EtOH/water and dried in vacuum. Yield: 217 mg (0.76 mmol, 42%); yellow solid of mp 99–100 °C; *υ*_max_(ATR)/cm^−1^ 3099, 3052, 2966, 2934, 2876, 2205, 1593, 1582, 1567, 1506, 1484, 1467, 1442, 1429, 1399, 1370, 1361, 1327, 1312, 1260, 1245, 1227, 1193, 1175, 1158, 1131, 1114, 1089, 1023, 991, 918, 897, 886, 857, 827, 801, 761, 744, 734, 716, 698, 688; ^1^H NMR (300 MHz, DMSO-d_6_) *δ* 0.84 (3 H, t, J = 7.3 Hz), 1.7–1.9 (2 H, m), 4.19 (2 H, t, J = 7.0 Hz), 6.5–6.6 (1 H, m), 7.5–7.6 (2 H, m), 7.67 (1 H, d, J = 8.7 Hz), 7.86 (1 H, d, J = 8.7 Hz), 8.1–8.2 (1 H, m), 8.19 (1 H, s), 8.24 (1 H, s), 8.5–8.6 (1 H, m), 8.96 (1 H, s); ^13^C NMR (75.5 MHz, DMSO-d_6_) *δ* 11.1, 23.2, 47.2, 101.9, 102.6, 110.5, 118.3, 122.2, 123.5, 123.9, 124.6, 128.1, 130.6, 132.8, 137.1, 146.3, 146.5, 149.2; HRMS for C_19_H_18_N_3_ [M^+^ + H] calcd. 288.14952, found 288.14914.

##### 3-[1-Cyano-2-(1-propargylindol-5-yl)-(*Z*)-ethenyl)]-3-pyridine (**2e**)

1-Propargylindol-5-carboxaldehyde (366 mg, 2.0 mmol) was dissolved in ethanol (p.a., 5 mL) and 3-pyridineacetonitrile (236 mg, 2.0 mmol) was added. A catalytic amount of piperidine (eight drops via a Pasteur pipette) was added and the reaction mixture was vigorously stirred under reflux for 24 h. After cooling to room temperature, the solvent was evaporated, and the residue was recrystallized from CH_2_Cl_2_/*n*-hexane. Yield: 380 mg (1.34 mmol, 67%); yellow solid of mp 183–184 °C; *υ*_max_(ATR)/cm^−1^ 3143, 2212, 1588, 1512, 1487, 1451, 1428, 1419, 1392, 1372, 1355, 1334, 1294, 1264, 1185, 1145, 1093, 1052, 1040, 1022, 1003, 948, 928, 911, 882, 863, 804, 774, 760, 730, 702, 682; ^1^H NMR (500 MHz, DMSO-d_6_) *δ* 3.4–3.5 (1 H, m), 5.17 (2 H, s), 6.6–6.7 (1 H, m), 7.5–7.6 (2 H, m), 7.70 (1 H, d, J = 8.7 Hz), 7.89 (1 H, d, J = 8.7 Hz), 8.1–8.2 (1 H, m), 8.21 (1 H, s), 8.26 (1 H, s), 8.5–8.6 (1 H, m), 8.97 (1 H, s); ^13^C NMR (125.8 MHz, DMSO-d_6_) *δ* 35.3, 75.9, 78.9, 102.7, 103.2, 110.7, 118.2, 122.6, 123.3, 123.9, 125.3, 128.4, 130.2, 130.5, 132.9, 136.7, 146.1, 146.5, 149.3; HRMS for C_19_H_14_N_3_ [M^+^ + H] calcd. 284.11822, found 284.11793.

#### 4.3.2. Synthesis of (*p*-Cymene)dichloridoruthenium(II) Complexes **3**

##### Complex **3a**

**2a** (59 mg, 0.23 mmol) was dissolved in CH_2_Cl_2_ (5 mL) and [Ru(*p*-cymene)Cl_2_]_2_ (70 mg, 0.11 mmol) was added. The reaction mixture was stirred at room temperature for 3 h. Ethyl acetate/*n*-hexane (1:4, 50 mL) was added and the formed precipitate was collected, washed with *n*-hexane and dried in vacuum. Yield: 82 mg (0.14 mmol, 61%); amber solid of mp 228–229 °C (dec.); *υ*_max_(ATR)/cm^−1^ 3104, 3059, 2961, 2931, 2872, 2213, 1585, 1567, 1486, 1470, 1427, 1387, 1331, 1308, 1276, 1248, 1192, 1158, 1101, 1083, 1057, 1030, 915, 870, 831, 808, 760, 726, 689, 667; ^1^H NMR (300 MHz, DMSO-d_6_) *δ* 1.32 (6 H, d, J = 6.9 Hz), 2.13 (3 H, s), 2.9–3.1 (1 H, m), 3.81 (3 H, s), 5.26 (2 H, d, J = 6.0 Hz), 5.47 (2 H, d, J = 6.0 Hz), 6.57 (1 H, s), 7.10 (1 H, s), 7.3–7.4 (2 H, m), 7.64 (1 H, s), 7.86 (1 H, d, J = 8.8 Hz), 7.9–8.0 (1 H, m), 8.18 (1 H, s), 8.97 (1 H, d, J = 5.6 Hz), 9.33 (1 H, s); ^13^C NMR (75.5 MHz, CDCl_3_) *δ* 18.3, 22.3, 30.7, 33.1, 82.3, 82.8, 97.3, 102.8, 103.7, 110.0, 123.3, 124.3, 124.5, 124.7, 128.7, 134.8, 138.4, 147.8, 151.8, 153.9; *m*/*z* (%) 306 (2), 271 (4), 258 (97), 243 (77), 216 (37), 134 (37), 119 (100), 91 (30).

##### Complex **3b**

**2b** (32 mg, 0.114 mmol) was dissolved in CH_2_Cl_2_ (5 mL) and [Ru(*p*-cymene)Cl_2_]_2_ (35 mg, 0.057 mmol) was added. The reaction mixture was stirred at room temperature for 3 h. Ethyl acetate/*n*-hexane (1:4, 50 mL) was added and the formed precipitate was collected, washed with *n*-hexane and dried in vacuum. Yield: 50 mg (0.086 mmol, 75%); amber solid of mp 143 °C (dec.); *υ*_max_(ATR)/cm^−1^ 3040, 2966, 2872, 2210, 1589, 1566, 1483, 1469, 1422, 1398, 1349, 1328, 1280, 1233, 1218, 1192, 1158, 1110, 1088, 1054, 1033, 1004, 941, 909, 875, 833, 807, 759, 717, 691, 654; ^1^H NMR (300 MHz, CDCl_3_) *δ* 1.31 (6 H, d, J = 6.9 Hz), 1.48 (3 H, t, J = 7.3 Hz), 2.13 (3 H, s), 2.9–3.1 (1 H, m), 4.19 (2 H, q, J = 7.3 Hz), 5.26 (2 H, d, J = 6.0 Hz), 5.47 (2 H, d, J = 6.0 Hz), 6.5–6.6 (1 H, m), 7.1–7.2 (1 H, m), 7.3–7.4 (2 H, m), 7.64 (1 H, s), 7.8–7.9 (1 H, m), 7.9–8.0 (1 H, m), 8.19 (1 H, s), 8.9–9.0 (1 H, m), 9.34 (1 H, s); ^13^C NMR (75.5 MHz, CDCl_3_) *δ* 15.5, 18.3, 22.3, 30.7, 41.3, 82.3, 82.7, 97.2, 101.8, 102.9, 103.7, 109.9, 123.1, 124.2, 124.5, 124.8, 128.9, 132.9, 134.8, 137.4, 147.8, 151.8, 153.8; ESI-MS *m*/*z* (%) 312.01, (45), 274.13 (100).

##### Complex **3c**

**2c** (39 mg, 0.114 mmol) was dissolved in CH_2_Cl_2_ (5 mL) and [Ru(*p*-cymene)Cl_2_]_2_ (35 mg, 0.057 mmol) was added. The reaction mixture was stirred at room temperature for 3 h. Ethyl acetate/*n*-hexane (1:4, 50 mL) was added and the formed precipitate was collected, washed with *n*-hexane and dried in vacuum. Yield: 60 mg (0.093 mmol, 82%); amber solid of mp 139–140 °C (dec.); *υ*_max_(ATR)/cm^−1^ 3039, 2962, 2932, 2873, 2213, 1588, 1568, 1524, 1471, 1451, 1389, 1346, 1277, 1226, 1181, 1160, 1111, 1088, 1057, 1031, 1005, 909, 869, 903, 749, 693; ^1^H NMR (300 MHz, CDCl_3_) *δ* 1.32 (6 H, d, J = 6.9 Hz), 1.38 (3 H, t, J = 7.2 Hz), 2.20 (3 H, s), 2.9–3.1 (1 H, m), 4.25 (2 H, q, J = 7.2 Hz), 5.27 (2 H, d, J = 6.0 Hz), 5.48 (2 H, d, J = 6.0 Hz), 7.4–7.5 (2 H, m), 7.64 (1 H, s), 7.9–8.0 (2 H, m), 8.03 (1 H, s), 8.9–9.0 (1 H, m), 9.33 (1 H, s); ^13^C NMR (75.5 MHz, CDCl_3_) *δ* 15.0, 18.3, 22.3, 30.7, 39.4, 82.3, 82.8, 97.2, 99.5, 103.7, 103.9, 110.0, 110.1, 121.2, 123.9, 124.0, 124.4, 125.0, 125.7, 132.3, 134.9, 146.6, 151.8, 154.2; HRMS for C_28_H_27_N_3_Cl_3_Ru [M^+^-Cl] calcd. 312.03086, found 312.03003.

##### Complex **3d**

**2d** (33 mg, 0.114 mmol) was dissolved in CH_2_Cl_2_ (5 mL) and [Ru(*p*-cymene)Cl_2_]_2_ (35 mg, 0.057 mmol) was added. The reaction mixture was stirred at room temperature for 3 h. Ethyl acetate/*n*-hexane (1:4, 50 mL) was added and the formed precipitate was collected, washed with *n*-hexane and dried in vacuum. Yield: 55 mg (0.093 mmol, 82%); amber solid of mp 216–217 °C (dec.); *υ*_max_(ATR)/cm^−1^ 3041, 2964, 2934, 2875, 2209, 1601, 1584, 1563, 1482, 1469, 1432, 1397, 1324, 1310, 1252, 1229, 1214, 1154, 1115, 1090, 1057, 1034, 1004, 912, 869, 826, 798, 772, 738, 687; ^1^H NMR (300 MHz, CDCl_3_) *δ* 0.93 (3 H, t, J = 7.3 Hz), 1.31 (6 H, d, J = 6.9 Hz), 1.8–1.9 (2 H, m), 2.13 (3 H, s), 2.9–3.1 (1 H, m), 4.09 (2 H, t, J = 7.0 Hz), 5.26 (2 H, d, J = 5.9 Hz), 5.46 (2 H, d, J = 5.9 Hz), 6.5–6.6 (1 H, m), 7.1–7.2 (1 H, m), 7.3–7.4 (2 H, m), 7.64 (1 H, s), 7.8–7.9 (1 H, m), 7.9–8.0 (1 H, m), 8.19 (1 H, s), 8.9–9.0 (1 H, m), 9.34 (1 H, s); ^13^C NMR (75.5 MHz, CDCl_3_) *δ* 11.4, 18.3, 22.3, 23.6, 30.7, 48.3, 82.3, 82.7, 97.2, 101.7, 102.8, 103.7, 110.1, 123.1, 124.2, 124.4, 124.8, 128.8, 129.7, 132.9, 134.8, 137.8, 147.8, 151.8, 153.8; HRMS for C_29_H_31_N_3_ClRu [M^+^-Cl] calcd. 558.12445, found 558.12401.

##### Complex **3e**

**2e** (32 mg, 0.114 mmol) was dissolved in CH_2_Cl_2_ (5 mL) and [Ru(*p*-cymene)Cl_2_]_2_ (35 mg, 0.057 mmol) was added. The reaction mixture was stirred at room temperature for 3 h. Ethyl acetate/*n*-hexane (1:4, 50 mL) was added and the formed precipitate was collected, washed with *n*-hexane and dried in vacuum. Yield: 49 mg (0.083 mmol, 73%); amber solid of mp >190 °C (dec.); *υ*_max_(ATR)/cm^−1^ 3221, 3093, 3037, 2964, 2873, 2209, 1586, 1568, 1481, 1425, 1398, 1324, 1305, 1271, 1194, 1142, 1115, 1094, 1057, 911, 870, 827, 803, 765, 734, 686; ^1^H NMR (300 MHz, CDCl_3_) *δ* 1.31 (6 H, d, J = 6.9 Hz), 2.13 (3 H, s), 2.4–2.5 (1 H, m), 2.9–3.1 (1 H, m), 4.89 (2 H, s), 5.27 (2 H, d, J = 6.1 Hz), 5.48 (2 H, d, J = 6.1 Hz), 6.1–6.2 (1 H, m), 7.2–7.3 (2 H, m), 7.46 (1 H, d, J = 8.7 Hz), 7.65 (1 H, s), 7.8–7.9 (1 H, m), 7.9–8.0 (1 H, m), 8.20 (1 H, s), 8.9–9.0 (1 H, m), 9.34 (1 H, s); ^13^C NMR (75.5 MHz, CDCl_3_) *δ* 19.3, 22.3, 30.7, 36.1, 74.2, 82.3, 82.8, 97.2, 102.4, 103.7, 110.1, 123.6, 124.3, 124.6, 125.1, 129.1, 132.7, 134.9, 137.4, 147.5, 151.8, 154.0; ESI-MS m/z (%) 312.01 (55), 284.12 (100).

### 4.4. Anticancer Activity

#### 4.4.1. Cell Line and Culture Conditions

518A2 melanoma (Department of Radiotherapy, Medical University of Vienna, Austria)^1^, MCF-7/Topo multi drug resistant (MDR) breast carcinoma, MCF-7 (ACC-115) breast carcinoma, HCT-116 (ACC-581) and HCT-116 p53-knockout colon carcinoma, Huh-7 (JCRB#0403) hepatocellular carcinoma, and EaHy.926 (ATCC^®^ CRL-2922^TM^) endothelial hybrid cells, FLO-1 and SK-GT-4 esophageal adenocarcinoma cells (Sigma-Aldrich, St. Louis, MO, USA) were cultivated in Dulbecco’s Modified Eagle Medium (DMEM) supplemented with 10% fetal bovine serum (20% for HDFa cells), and 1% antibiotic-antimycotic or 1 % ZellShield^®^ at 37 °C, 5% CO_2_, and 95% humidity. To keep MCF-7/Topo cells resistant, topotecan was added to the cell culture medium 24 h after every passage. Cells were grown at 5% CO_2_, and 95% humidity. Only mycoplasma-free cell cultures were used.

#### 4.4.2. MTT Assay

The water-soluble tetrazolium salt 3-(4,5-dimethylthiazol-2-yl)-2,5- diphenyltetrazolium bromide (= MTT) was used to identify viable cells, which reduce it to violet formazan crystals. Therefore, the cancer and hybrid cells (5 × 10^4^ cells/mL, 100 μL/well) were grown in 96-well plates for 24 h under cell culture conditions. After the treatment with various concentrations (100 μM–0.5 nM) of test compounds **2a**–**e** and **3a**–**e**, positive controls sorafenib and gefitinib, and DMSO for 72 h, 12.5 μL of a 0.5% MTT solution in PBS were added to the cells and incubated for another 2 h at 37 °C. Subsequently the microplates were centrifuged (300 g, 5 min, 4 °C), the supernatant medium was discarded, and the formazan dissolved in 25 μL of DMSO containing 10% SDS and 0.6% acetic acid for at least 1 h (37 °C) in the dark. The absorbance of formazan (λ: 570 nm), and the background (λ: 630 nm) was measured with a microplate reader (Tecan infinite F200). The IC_50_ values were derived from dose-inhibition curves as the means ± SD of four independent experiments with respect to vehicle treated control cells set to 100%. For these data the “log (inhibitor) vs. normalized response-Variable slope” curve-fitting function was used (GraphPad Prism9).

#### 4.4.3. Hexosaminidase Assay

5 × 10^3^ cells/well of esophageal adenocarcinoma cell lines (FLO-1 and SK-GT-4) were plated in 96-well plate and allowed to grow for 24 h in complete DMEM media. Following 24 h, cells were treated with increasing doses of **2a**, **2b** and **3a**. The cell viability was measured at 72 h using an enzymatic hexosaminidase assay [46]. The percentage of cell viability was calculated by comparison with the controls.

#### 4.4.4. Cell Cycle Analysis

HCT-116 and HCT-116p53-/- (p53 knockout mutant) colon carcinoma cells (1 × 10^5^ cells/mL, 3 mL/well) were seeded in 6-well cell culture plates for 24 h under cell culture conditions (37 °C, 5% CO_2_, 95% humidity). The treatment of compounds **2a**, **2b**, **3a**, sorafenib, and gefitinib (at their IC_50_ doses, or 50 μM for gefitinib in wildtype cells) or solvent (DMSO) was carried out for 24 h. The cells were separated through trypsinization, centrifuged (5 min, 300× *g*, 4 °C) and fixed in 70% EtOH for at least 24 h at 4 °C. Before flow-cytometric measurement the cells were washed with PBS and stained with propidium iodide solution (50 μg/mL PI, 0.1% sodium citrate, 50 μg/mL RNAse A in PBS, pH = 7.4). The DNA content of at least 10,000 single cells was measured with a Beckmann Coulter Cytomics FC500 flow cytometer (λ_em_: 570 nm, λ_ex_: 488 nm laser source) and the ratio of the cell cycle phases (sub-G1, G1, S, G2/M) were determined as means from duplicates by CXP software (Beckman Coulter).

#### 4.4.5. Ethidium Bromide Assay

In order to determine the intrinsic background fluorescence of the test compounds, 100 μL TE buffer were placed into wells of a black 96-well microplate followed by the addition of 11.1 µL of TE buffer solutions of the test compounds with increasing concentrations. In the remaining wells of the plate, 90 μL TE buffer were placed followed by the addition of 10 μL salmon sperm DNA (0.1 mg/mL) and 11.1 μL of the test compounds in order reach compound concentrations of 25 μM, 50 μM, 75 μM und 100 μM in the wells. Negative controls were treated with DMSO. The microplates were incubated at 37 °C for 2 h. Then, 100 μL/well of an EtBr solution (10 μg/mL in TE-Puffer) were added and the fluorescence was measured at 535 nm and 590 nm using a microplate reader (Tecan Infinite F200). The background fluorescence was subtracted from the measured fluorescence for the test compound/EtBr samples. The means of the three experiments plus the corresponding standard deviations were calculated, and negative controls were set to 100%.

#### 4.4.6. ROS Formation Assay

Cells of HCT116 wildtype and HCT116 p53-knockout cell lines were placed into 96-well plates at concentrations of 0.1 × 10^6^ cells/mL in volumes of 100 μL. The cells were incubated for 24 h, before the cells were treated with test compounds at 4-fold IC_50_ concentrations (200 μM for gefitinib in wildtype cells). After additional incubation for 24 h, the plates were centrifuged for 5 min at 300× and 4 °C, and the supernatant was discarded. Then, the cells were incubated for 4 h with 50 μL/well of 0.1% NBT solution. The plates were centrifuged (300× *g*, 5 min at 4 °C) and the liquids were carefully removed, before 50 μL/well of 2 M KOH and 65 μL/well DMSO were added, followed by 30 min incubation. Absorption was measured using a Tecan Infinite F200 plate reader at 630 nm and 405 nm. Experiments were carried out in quadruplicate.

#### 4.4.7. Caspase 3/7 Activation Assay

Caspase-3/7 activity was measured using the Cell Meter^TM^ Caspase 3/7 Activity Apoptosis Assay Kit (ATT Bioquest^®^). Therefore, HCT-116 and HCT-116p53-/- (p53 knockout mutant) colon carcinoma cells (1 × 10^5^ cells/mL, 67.5 μL/well) were grown under cell culture conditions (37 °C, 5% CO_2_, 95% humidity) in black 96-well plates for 24 h. The cells were incubated with **2a**, **2b**, **3a**, sorafenib, and gefitinib (2 × IC_50_ and 4 × IC_50_, or 100 and 200 μM of gefitinib in wildtype cells) or solvent (DMSO) for 24 h under cell culture conditions, following by addition of fluorogenic caspase-3/7 substrate solution and 60 min incubation at rt. The fluorescence intensity (λ_ex_: 485 nm, λ_em_: 521 nm) was measured using a microplate reader (Tecan infinite F200). The blank values without cells were subtracted to reduce the background signals of the in triplicated performed measurement. The results were quoted as means ± SEM.

#### 4.4.8. LDH Assay

Unspecific cytotoxicity induced by treatment with **2a**, **2b**, **3a**, sorafenib (LC Laboratories, Woburn, MA, USA) and gefitinib at their IC_50_ and ½ IC_50_ doses (50 and 25 μM for gefitinib in wildtype cells) was determined by measuring the release of lactate dehydrogenase (LDH) from HCT-116 wildtype and p53-knockout cells into the supernatant after 12 h (Cytotoxicity Detection KitPLUS LDH, Roche Diagnostics GmbH, Mannheim, Germany) [47]. The supernatant of treated samples was collected, and adherent untreated cells were lysed with 2% Triton X-100 for 10 min to determine the maximum LDH release. All samples were mixed with catalyst and dye solution for 30 min, resulting in the formation of formazan dye proportional to LDH enzyme activity. The absorbance was measured at 490/630 nm using an ELISA reader (Dynex Technologies, Denkendorf, Germany) and cytotoxicity was determined by subtracting the percentage of LDH release under control conditions of those from treated samples. Experiments were performed in duplicate, and data are given as mean percentage changes ± SD as compared to controls of n = 3 experiments.

#### 4.4.9. Colony Formation Assay

Cells at a concentration of 500 cells/well were seeded and incubated overnight. Test compounds (IC_50_ doses, or 20 μM for gefitinib in wildtype cells) were added followed by incubation for 5 d. DMSO served as negative control. After incubation, cells were washed twice with PBS and fixed with 100% EtOH for 30 sec. After treatment with 0.5% crystal violet for 3 min, cells were washed with water. The colonies (cell aggregates of at least 50 cells) were counted using a microscope [34]. The experiment was carried out in triplicate.

#### 4.4.10. Tumor Spheroid Inhibition Assay

The tumor spheroids were obtained following the hanging-drop method [48]. Drops with cells were incubated for 3 d (37 °C, 5% CO_2_, 95% humidity). The small cell aggregates were taken up carefully with a cut pipette tip and placed into agarose-coated wells of a 24-well plate. The wells were filled with medium (1 mL). Test compounds were added to the medium to reach end concentrations of IC_50_, 5 μM und 10 μM. The spheroids were incubated for 5 d, and their sizes were determined using a microscope. The experiment was performed in triplicate.

#### 4.4.11. Tube Formation Assay

EA.hy926 endothelial hybrid cells (100 μL/well, 5 × 10^5^ cells/mL) were seeded on thin layers of basement membrane-like matrix Matrigel^®^ (Corning, Corning, NY, USA) in μ-Slides (Ibidi, Gräfelfing, Germany) and cultivated for 24 h in EndoPrime low serum (Capricorn, Ebsdorfergrund, Germany) endothelial medium. The cells were treated with **2a**, **2b**, **3a**, gefitinib, sorafenib (all of them 5 μM), and combretastatin A-4 (10 nM) or solvent (DMSO) for 5 h under cell culture conditions (37 °C, 5% CO_2_, and 95% humidity), till tubular structures had been formed in the control wells. Anti-angiogenic effects were documented via light microscopy (Zeiss Axiovert 135, 100× magnification), in which the determination of the substances was carried out in duplicates. EA.hy926 cells were reviewed on their vitality via MTT-assay as described above. Therefore, a viability higher than 80% of negative control set to 100% were confirmed. The images were analysed with ImageJ (angiogenesis-analysing tool) and compared for the number of junctions, meshes, master segments (MS) and length of MS to quantify the tube formation process [49].

### 4.5. Molecular Docking

AutoDock Vina software (Molecular Graphics Lab, Scripps Research Institute, http://vina.scripps.edu/ (accessed on 28 April 2022)) was used to investigate the interaction of compounds **2a** and **2b** with the protein cavity of EGFR (PDB ID: 1M17) and VEGFR-2 (PDB ID: 2OH4) [50,51,52]. AutoDock tools were applied to prepare proteins and ligands for docking by using default parameters. Polar hydrogens and Kollman and Gasteiger charges were added to the protein and the ligand before docking. The grid box of 40 × 40 × 40 size was designed around the ligand binding domain. Lamarckian GA was used to determine the best protein-ligand conformations (approximately 10) for the analyses. The most stable ligand conformation in the protein was selected based on lower binding energy, and the number of hydrogen bonds. The ligand-protein complex was visualized using Pymol (https://pymol.org/2/ (accessed on 28 April 2022)) [53].

## Figures and Tables

**Table 1:** Inhibitory activities (IC_50_ values in μM after 72 h of incubation) ^1^ of compounds **2a**–**e** and **3a**–**e**. against MCF-7 breast carcinoma, HCT-116 wildtype (wt) and HCT-116 p53-negative colon carcinoma cells. Sorafenib, gefitinib and NAMI-A served as control compounds.

Compound	HCT-116 wt	HCT-116 p53-Negative	MCF-7
**2a**	3.0 ± 0.2	0.9 ± 0.2	1.7 ± 0.2
**2b**	0.4 ± 0.1	0.6 ± 0.1	2.6 ± 0.6
**2c**	7.7 ± 0.3	6.1 ± 0.5	8.4 ± 0.7
**2d**	>50	>50	>50
**2e**	>50	>50	>50
**3a**	0.02 ± 0.003	1.7 ± 0.6	0.5 ± 0.7
**3b**	4.0 ± 0.6	1.7 ± 0.6	2.7 ± 0.4
**3c**	>50	>50	3.2 ± 0.5
**3d**	>50	>50	>50
**3e**	>50	>50	>50
Sorafenib	10 ± 1.8	7.7 ± 0.7	9.1 ± 1.1
Gefitinib	>50	17.8 ± 3.7	21.1 ± 9.4
NAMI-A	>50	>50	>50

^1^ IC_50_ values ± standard deviation (SD) were calculated from the dose-response curves of four independent MTT experiments.

**Table 2:** Inhibitory activities (IC_50_ values in μM after 72 h of incubation) ^1^ of compounds **2a**, **2b** and **3a** against MCF-7/Topo breast carcinoma, 518A2 melanoma, EaHy.926 endothelial/lung carcinoma, Huh-7 hepatocellular carcinoma, FLO-1 and SK-GT-4 esophageal adenocarcinoma cells. Sorafenib and gefitinib served as positive controls.

Cell Line	2a	2b	3a	Sorafenib	Gefitinib
MCF-7/Topo	0.18 ± 0.09	1.5 ± 0.1	0.10 ± 0.02	7.4 ± 0.4	27.6 ± 2.4
518A2	1.4 ± 0.6	2.8 ± 0.3	0.6 ± 0.1	9.7 ± 2.1	27.5 ± 1.6
EaHy.926	1.3 ± 0.2	0.9 ± 0.1	1.9 ± 0.2	12.8 ± 0.2	9.9 ± 2.9
Huh-7	0.04 ± 0.01	-	0.01 ± 0.001	0.9 ± 0.08	>50
FLO-1	1.2 ± 0.2	>40	2.5 ± 0.3	-	-
SK-GT-4	>40	>40	>40	-	-

^1^ IC_50_ values ± standard deviation (SD) were calculated from the dose-response curves of three independent experiments.

**Table 3:** Binding energies (B.E.) and H bond information for **2a** and **2b** when docked into EGFR and VEGFR-2.

Protein	Compound	B.E. (kcal/mol)	H Bonds	Distance (Å)	Amino Acid (s)
EGFR	**2a**	−8.2	1	3.3	MET769
	**2b**	−7.7	2	3.3 3.3	GLU738 ASP831
VEGFR-2	**2a**	−9.5	2	2.8 3.5	ILE913 GLU915
	**2b**	−9.5	2	2.8 3.5	ILE913 GLU915

## Data Availability

Original data can be requested from the corresponding author.
